# Ketamine, Propofol, and the EEG: A Neural Field Analysis of HCN1-Mediated Interactions

**DOI:** 10.3389/fncom.2013.00022

**Published:** 2013-04-05

**Authors:** Ingo Bojak, Harry C. Day, David T. J. Liley

**Affiliations:** ^1^Centre for Computational Neuroscience and Cognitive Robotics, School of Psychology, University of BirminghamBirmingham, UK; ^2^Donders Centre for Neuroscience, Donders Institute for Brain, Cognition and Behaviour, Radboud University Nijmegen Medical CentreNijmegen, Netherlands; ^3^Brain and Psychological Sciences Research Centre, Faculty of Life and Social Sciences, Swinburne University of TechnologyHawthorn, VIC, Australia; ^4^Cortical Dynamics Ltd.Hawthorn, VIC, Australia

**Keywords:** ketamine, propofol, EEG, HCN1, neural field theory, drug interaction, anesthesia, infra-additivity

## Abstract

Ketamine and propofol are two well-known, powerful anesthetic agents, yet at first sight this appears to be their only commonality. Ketamine is a dissociative anesthetic agent, whose main mechanism of action is considered to be *N*-methyl-d-aspartate (NMDA) antagonism; whereas propofol is a general anesthetic agent, which is assumed to primarily potentiate currents gated by γ-aminobutyric acid type A (GABA_A_) receptors. However, several experimental observations suggest a closer relationship. First, the effect of ketamine on the electroencephalogram (EEG) is markedly changed in the presence of propofol: on its own ketamine increases θ (4–8 Hz) and decreases α (8–13 Hz) oscillations, whereas ketamine induces a significant shift to beta band frequencies (13–30 Hz) in the presence of propofol. Second, both ketamine and propofol cause inhibition of the inward pacemaker current *I*_h_, by binding to the corresponding hyperpolarization-activated cyclic nucleotide-gated potassium channel 1 (HCN1) subunit. The resulting effect is a hyperpolarization of the neuron’s resting membrane potential. Third, the ability of both ketamine and propofol to induce hypnosis is reduced in HCN1-knockout mice. Here we show that one can theoretically understand the observed spectral changes of the EEG based on HCN1-mediated hyperpolarizations alone, without involving the supposed main mechanisms of action of these drugs through NMDA and GABA_A_, respectively. On the basis of our successful EEG model we conclude that ketamine and propofol should be antagonistic to each other in their interaction at HCN1 subunits. Such a prediction is in accord with the results of clinical experiment in which it is found that ketamine and propofol interact in an infra-additive manner with respect to the endpoints of hypnosis and immobility.

## Introduction

Ketamine, a phenylcyclohexylpiperidine (PCP) derivative, is a powerful psychoactive drug that is predominantly used as a sedative and general anesthetic agent in humans and animals (Sinner and Graf, 2008). Ketamine occurs as two stereoisomers, R(−) and S(+), in which the latter is found to be some three to four times more potent (White et al., 1985), but despite such differences in potency the drug is generally made available clinically as a racemate (racemic mixture) that contains both stereoisomers in equal proportion. Ketamine is classified as a dissociative agent due to its ability to induce hallucinations and perceptual/environmental detachment (Wolff and Winstock, 2006). Because of these properties it has become popular recreationally. At sufficiently high doses it has been reported to induce a state of dissociation comparable to that of schizophrenia, and as a consequence has found use as a pharmacological model for psychosis (Bubenikova-Valesova et al., 2008; Corlett et al., 2011). More recently its therapeutic use has been re-evaluated in light of evidence suggesting that sub-anesthetic doses may aid in the treatment of bipolar affective disorder and major depression (Mathew et al., 2012; Murrough, 2012; Murrough et al., 2012).

**Figure 1:**
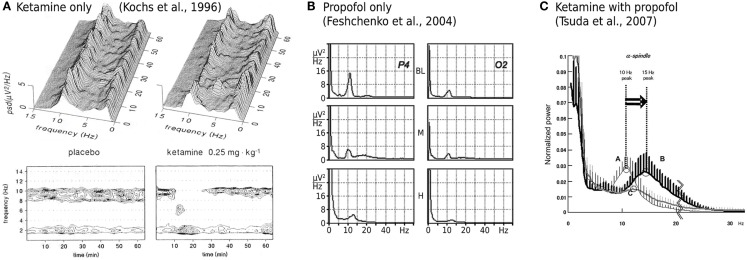
**Experimentally described EEG power spectral changes induced by ketamine and propofol**. **(A)** A single bolus dose of ketamine (0.25 mg/kg) is associated with resting α activity being transiently replaced by θ band activity. Data shown is the mean power spectral density (PSD) of EEG recorded in three subjects from a Cz-A1/A2 (vertex-linked ears) montage. Figure adapted and used with permission from Kochs et al. (1996). **(B)** Average spectra of EEG recorded during two sequential target concentrations of propofol in a single subject. BL = baseline, *M* = 1.25 μg/ml propofol, *H* = 2.5 μg/ml propofol. α band EEG recorded from parietal (P4) and occipital (O2) electrodes reveals minimal changes in peak frequency with increasing propofol concentration. At medium propofol concentrations (*M*) the α rhythm shifts to central and frontal areas (figure not shown) without any significant change in frequency. Figure adapted and used with permission from Feshchenko et al. (2004). **(C)** Fifteen minutes after the administration of a ketamine bolus (1 mg/kg; bold line labeled B), in the presence of a steady state target controlled propofol level (3.5 μg/ml; thin line labeled A), peak α band EEG activity is markedly shifted to higher frequencies. Data shown is mean PSD recorded at Fp1-A1, with an Fpz reference, from nine subjects undergoing elective abdominal surgery. Figure used with permission from Tsuda et al. (2007).

While ketamine is widely believed to act principally through the non-competitive antagonism of *N*-methyl-d-aspartate (NMDA) receptor mediated glutamatergic activity (Irifune et al., 1992; Oye et al., 1992), two significant pieces of empirical evidence have emerged that challenge such a unitary view. Firstly, dizocilpine (also known as MK801), an even more potent non-competitive NMDA antagonist, produces no significant hypnotic effect (Kelland et al., 1993; Irifune et al., 2007). Secondly, ketamine’s effect on spontaneous electroencephalogram (EEG) activity is qualitatively altered when administered in the presence of propofol, a widely used intravenous general anesthetic agent that, at clinically meaningful concentrations, has little or no effect on NMDA mediated currents. Ketamine alone has been shown to reduce spectral edge frequencies, an effect that is driven predominately by increases in absolute θ band (4–8 Hz) power at the expense of α band (8–13 Hz) power (Schuttler et al., 1987; Kochs et al., 1996), see Figure 1A. In contrast, ketamine administered in the presence of steady state propofol levels is associated with a definite acceleration of α band activity; increasing its peak frequency by up to 4.7 Hz (Hayashi et al., 2007; Tsuda et al., 2007), see Figure 1C. Propofol on its own roughly maintains the α peak frequency with an anteriorization of power (decrease occipital, increase frontal), see Figure 1B; though an additional broadband “beta buzz” just above α frequencies, “biphasic” response dynamics and smooth transitions to lower frequencies can confound the picture (Schwender et al., 1996; Kuizenga et al., 1998, 2001; Feshchenko et al., 2004; Breshears et al., 2010; Cimenser et al., 2011). We assume here from previous theoretical studies (Liley et al., 2003; Hutt and Schimansky-Geier, 2008; Hutt and Longtin, 2010; Hindriks and van Putten, 2012) that these complications can be accounted for by mechanisms not considered in this work, in particular the prominent γ-aminobutyric acid type A (GABA_A_) agonism of propofol that affects dominantly inhibitory postsynaptic currents (Kitamura et al., 2003). Furthermore, the acceleration due to ketamine observed by Hayashi et al. (2007) and Tsuda et al. (2007) that we wish to describe occurred on top of a clear α rhythm at steady propofol concentration, see Figure 1C. Thus we assume in the following that the action of propofol is largely neutral concerning the α peak frequency (while unspecified concerning total α band spectral power).

Recently a number of alternative, behaviorally relevant, molecular targets for ketamine action have been identified (Schnoebel et al., 2005; Hevers et al., 2008; Chen et al., 2009). Of particular significance is the identification of hyperpolarization-activated cyclic nucleotide-gated (HCN) potassium channel subunits as a target for ketamine action (Chen et al., 2009). HCN subunits, of which there are four isoforms (HCN1–4), assemble to form a tetrameric ion channel that mediates an inward (i.e., depolarizing) hyperpolarization-activated pacemaker current *I*_h_ implicated in neuronal rhythmogenesis (Biel, 2009; Biel et al., 2009). In particular the HCN1 isoform has been identified as a molecular substrate for the actions of ketamine (Chen et al., 2009): ketamine causes inhibition of HCN1-mediated *I*_h_ currents, and hence membrane hyperpolarization, in pyramidal neurons from wild-type but not HCN1-knockout mice. The potency of ketamine to provoke a loss of the righting reflex (the ability to regain footing from a back position), which is a behavioral correlate of hypnosis, is also strongly reduced in HCN1-knockout mice. Hence a causal relationship between ketamine-induced membrane hyperpolarization and its clinical effects can be made. The existence of such a causal relationship is made more likely by evidence indicating that the hypnotic potency of propofol is also reduced in HCN1-knockout mice, in approximate proportion to its ability to inhibit HCN1-mediated membrane depolarization (Chen et al., 2009).

It should be noted though that the hypnotic response was not abolished entirely in HCN1-knockout mice by either ketamine or propofol (Chen et al., 2009), thus other effects like the mentioned GABA_A_ agonism will be required to fully understand the hypnotic action of these agents. However, etomidate, which has no effect on HCN1 channels, showed no loss of hypnotic effect in HCN1-knockout mice (Chen et al., 2009), suggesting the specific involvement of HCN1-mediated *I*_h_ currents for ketamine and propofol. The identification of a shared molecular target for ketamine and propofol action could offer a new possibility to account for the qualitatively disparate electroencephalographic effects of ketamine alone and in the presence of propofol. On this basis we hypothesized that by modeling the differential effects of ketamine and propofol on neuronal membrane hyperpolarization, in the context of an established theory of resting EEG (Liley et al., 2002, 2010, 2011; Bojak and Liley, 2005), we would be able to describe the observed effects on the EEG at least qualitatively. Because current depth of anesthesia monitoring approaches are either insensitive (Faraoni et al., 2009; Nonaka et al., 2012), or respond anomalously (Hans et al., 2005; Sengupta et al., 2011), to the hypnotic effects of ketamine, understanding the mechanism by which ketamine and propofol interact electroencephalographically will ultimately assist in the development of improved approaches to clinically monitor the hypnotic effects of combinations of these drugs. The combination of propofol and ketamine (often referred to as *ketofol*) is becoming increasingly important in the procedural sedation setting where rapid and effective sedation and analgesia, with minimal cardiorespiratory/hemodynamic compromise, is required (Hui et al., 1995; Frizelle et al., 1997; Sakai et al., 1999; Phillips et al., 2010).

## Materials and Methods

### Modeling drug response and interactions

The simplest pharmacodynamic model of drug effect involving two or more agonists is that of competitive ligand-receptor binding. It is easily shown for two full agonists competing for the same receptor binding site, that the fractional receptor occupancy θ, as a function of the respective drug concentrations (*D*_1_, *D*_2_) is (Shafer et al., 2008)

(1) $$\begin{array}{ll} \theta & =\frac{k_{2}D_{1}+k_{1}D_{2}}{k_{2}D_{1}+k_{1}D_{2}+k_{1}k_{2}},\;\text{or} \end{array}$$

(2) $$\begin{array}{ll} \frac{\theta }{1-\theta } & =\frac{D_{1}}{k_{1}}+\frac{D_{2}}{k_{2}}. \end{array}$$

For *D*_1_ → ∞ and/or *D*_2_ → ∞, one then finds þeta → 1, i.e., full receptor occupancy. *k*_1_, *k*_2_ > 0 are the respective drug-receptor dissociation constants, which are equivalent to single drug concentrations that produce 50% receptor occupancy, i.e., þeta = 1/2. In general a pharmacodynamic effect *E* is assumed to be some monotonic function of θ, i.e., *E* = *f*(θ) ≡ *g*(*D*_1_, *D*_2_). For a fixed effect *E* the locus of points (*D*_1_, *D*_2_) defines a *response isobole* and *E* = *g*(*D*_1_, *D*_2_) a *response surface*. Now consider the case of competitive binding and drug interaction (Greco et al., 1995)

(3) $$\frac{\theta }{1-\theta }=\frac{D_{1}}{k_{1}}+\frac{D_{2}}{k_{2}}+\frac{\eta D_{1}D_{2}}{k_{1}k_{2}},$$

where η defines an interaction term. It can be easily demonstrated that

(4) $$\begin{array}{cccccc} \frac{\theta -1}{\theta } & < & \eta & < & 0 & \Rightarrow \;\text{ infra-additivity / antagonism,}\\ & & \eta & = & 0 & \Rightarrow \;\text{ additivity,}\\ & & \eta & > & 0 & \Rightarrow \;\text{ synergy}\text{.} \end{array}$$

Inspired by these considerations, we chose here to describe the general pharmacodynamic effect of our two ligands by the following bilinear form

(5) $$E=c_{1}D_{1}+c_{2}D_{2}+c_{12}D_{1}D_{2}.$$

This ansatz represents the simplest extension beyond the purely additive; and the sign of *c*_12_ then has the same interpretation as the sign of η in Eq. 4. We will use this bilinear form below to parameterize the dependence of the induced hyperpolarizations on normalized concentrations of propofol and ketamine, respectively.

One can however relate Eqs 3 and 5 more directly. Assume first that the pharmacodynamic effect is directly proportional to receptor occupancy, i.e., *E* ∝ θ. Then *k*_1_ and *k*_2_ become the respective “half maximum effective concentrations” (EC50s) at which 50% of the maximum response is observed for each drug applied alone. Furthermore, assume that the receptor occupancy remains relatively small $\theta \approx \theta ∕\left(1-\theta \right)=D_{1}∕k_{1}+D_{2}∕k_{2}+\eta D_{1}D_{2}∕\left(k_{1}k_{2}\right)<1∕2$, so that the effect *E* < *E*_max_/2. The half-maximal inhibition of HCN1 subunit-mediated ionic currents by racemic ketamine occurs at a concentration of approximately 16 μM (Chen et al., 2009), which is significantly greater than the estimated minimum free plasma concentrations of 2.9 μM required to produce surgical anesthesia in humans (Grant et al., 1983). Data for the half-maximal inhibition of HCN1-mediated ionic currents by propofol is to our knowledge not available. However, because HCN1-knockout mice are significantly less sensitive to the effects of propofol than wild-type ones, we can speculate that the ED50 (the “half maximum effective dose”) for unresponsiveness with propofol in wild-type mice corresponds roughly to the half maximum of the neuronal changes (EC50). Chen et al. (2009) found this to be approximately 7 mg/kg. Using the volume of distribution of 1.38 l/kg (Cox et al., 1998) in the rat (murine values not available), EC50 is then about 5.1 mg/l or 29 μM, which is significantly greater than the minimum free plasma concentration ∼8.5 μM for surgical anesthesia. Thus $D_{\text{ketamine}}∕k_{\text{ketamine}}+D_{\text{propofol}}∕k_{\text{propofol}}+\eta D_{\text{ketamine}}D_{\text{propofol}}∕\left(k_{\text{propofol}}k_{\text{ketamine}}\right)<1∕2$ is approximately satisfied as long as $\eta <\frac{1}{2}.$ While the *E*_max_ for ketamine-induced membrane hyperpolarization in murine pyramidal neurons is of the order of −4 mV, the actual value of *E*_max_ will depend on the species and the recording conditions/preparation. In the absence of any information to the contrary one can assume that *E* < *E*_max_/2. Thus our ansatz Eq. 5 can be considered as following from Eq. 3 under a range of reasonable assumptions.

### Liley model and eigenspectrum calculation

We base our investigation in this paper on the Liley et al. (2002) model, which is a typical neural field model (Deco et al., 2008; Coombes, 2010; Bressloff, 2012; Liley et al., 2012). In Bojak and Liley (2005) 73,454 different parameter sets, which produce biologically plausible resting state activity, were found for this model. We use here also the “eigenspectrum” approach introduced in Bojak and Liley (2005) to directly predict EEG power spectral densities (PSDs) from a given parameter set. In the following we will briefly review a few key features of the Liley et al. (2002) model and of the eigenspectrum approach that will play a role for the analysis in this paper, and refer the reader to the original reference for more detail. The Liley et al. (2002) model can be written concisely as follows:

(6) $$\begin{array}{l} \tau_{k}\frac{\partial }{\partial t}h_{k}\left(x,t\right)=h_{k}^{r}-h_{k}\left(x,t\right)+\underset{l=e,i}{\sum }\frac{h_{lk}^{eq}-h_{k}\left(x,t\right)}{\left|h_{lk}^{eq}-h_{k}^{r}\right|}I_{lk}\left(x,t\right), \end{array}$$

(7) $$\begin{array}{lll} {\left(\frac{1}{\gamma_{lk}}\frac{\partial }{\partial t}+1\right)}^{2}I_{lk}\left(x,t\right) & & \\ & =\frac{\Gamma_{lk}e}{\gamma_{lk}}\left\{\frac{N_{lk}^{\beta }S_{l}^{\max}}{1+e^{\sqrt{2}\left[h_{l}\left(x,t\right)-\mu_{l}\right]/\sigma_{l}}}+\Phi_{lk}\left(x,t\right)+p_{lk}\left(x,t\right)\right\}, \end{array}$$

(8) $$\begin{array}{c} \left[{\left(\frac{1}{v_{lk}\Lambda_{lk}}\frac{\partial }{\partial t}+1\right)}^{2}-\frac{3}{2}\frac{1}{\Lambda_{lk}^{2}}\nabla^{2}\right]\Phi_{lk}\left(x,t\right) \end{array}\begin{array}{c} \;=\frac{N_{lk}^{\alpha }S_{l}^{\max}}{1+e^{\sqrt{2}\left[h_{l}\left(x,t\right)-\mu_{l}\right]/.\sigma_{l}}}. \end{array}$$

In all these equations *l*, *k* = *e*, *i* serve as indices for excitatory and inhibitory neural populations, respectively, and **x** gives their position on a two-dimensional cortical sheet. The mean excitatory soma membrane potential *h_e_*(**x**, *t*) of Eq. 6 is taken to predict the EEG. In the absence of postsynaptic inputs these potentials *h_k_*(**x**, *t*) decay to their resting values $h_{k}^{r}$. The inputs *I_lk_*(**x**, *t*) correspond to postsynaptic potentials and are weighted by ionic driving forces $h_{lk}^{\text{eq}}-h_{k}\left(x,t\right)$, where the $h_{lk}^{\text{eq}}$ are the respective Nernst potentials. These weights are normalized at rest to +1 (excitatory) and −1 (inhibitory), respectively. A postsynaptic input in Eq. 7 uses double indices to indicate source and target (for example, *I_ei_*(**x**, *t*) is excitatory input to an inhibitory neural population). Γ*_lk_* is the mean peak amplitude induced by a single presynaptic pulse δ(*t* − *t*_p_), and 1/γ*_lk_* the corresponding rise time to this peak of a postsynaptic “α form” response *I*(**x**, *t*) ∝ γ^2^*te*^−γ*t*^Θ(*t* − *t*_p_), where Θ is the Heaviside step function and δ the Dirac delta function. Extra-cortical input is given by *p_lk_*(**x**, *t*), and is here assumed to be shaped noise (*p_ee_*), static (*p_ei_*), or absent (*p_ik_*). The noise represents the average of uncorrelated input to the many neurons in the neural mass. For simplicity it is imposed only on the excitatory extra-cortical input to excitatory neurons, which is sufficient to generate the full dynamical range of the model. Finally, activity is propagated cortico-cortically via Eq. 8 with a standard damped wave equation (Jirsa and Haken, 1996; Robinson et al., 1997). The activity propagation through Φ*_lk_*(**x**, *t*) represents a synaptic footprint which falls off exponentially with characteristic distance scale Λ*_lk_*, and fibers having conduction velocity ν*_lk_*. Since there are no long-range inhibitory fibers, we can set Φ*_ik_* ≡ 0 in the following. Short range connectivity is both excitatory and inhibitory, and is represented by the first term in the curly brackets of Eq. 7. Note that Eq. 8 can be improved upon (Bojak and Liley, 2010), but its main role is in this case to include a larger variety of EEG wavelengths as will become apparent. Our main conclusions are not affected even for the radical choice of an entirely homogeneous cortex, i.e., upon removing all spatial dependence.

The eigenspectrum approach (Bojak and Liley, 2005) assumes that Eqs 6–8 have a “fixed point” solution for a homogeneous cortex with static *p_ee_*. All variables are then linearly expanded around this solution, and auxiliary variables ${Ĩ}_{lk}=\partial I_{lk}∕\partial t$ and ${\tilde{\Phi }}_{ek}=\partial \Phi_{ek}∕\partial t$ are used to turn Eqs 6–8 into 14 first order ODEs. One can then Fourier-transform in space and time, and obtains an equation for the 14-dimensional state vector **s** in the form

(9) $$i\omega \text{s}(\omega ,\text{k})={\text{J}(\text{k}}^{\text{2}}\text{)}\cdot \text{s}(\omega ,\text{k})+\text{P}(\omega ,\text{k}),$$

where **J** is the Jacobian matrix and **P**(ω, **k**) contains the remainder of the extra-cortical input, i.e., the variation of *p_ee_* with subtracted mean. Note that the only spatial derivative here is the Laplacian in Eq. 8, hence the Fourier-transformed Jacobian is a function of the square of wavenumber **k**. One can then show (Bojak and Liley, 2005) that

(10) $${\left|h_{e}\left(\omega ,k\right)\right|}^{2}={\left|\overset{14}{\underset{n=1}{\sum }}\frac{c_{n}\left(k\right)}{i\omega -\lambda_{n}\left(k\right)}\right|}^{2},$$

where both the coefficients *c_n_* and the eigenvalues λ*_n_* can be obtained from a decomposition of the Jacobian in both left and right eigenmatrices, and *k* ≡ |**k**|.

**Figure 2:**
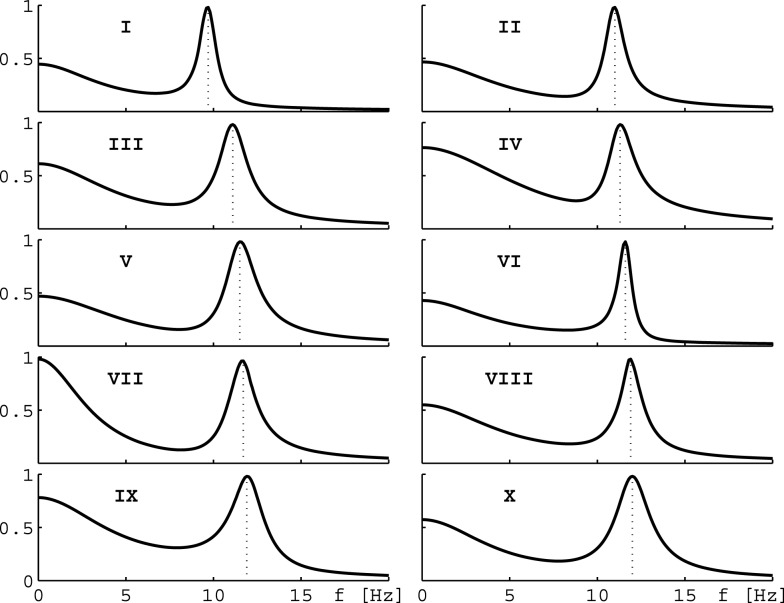
**Eigenspectra of 10 parameter sets**. The panels show eigenspectra estimated with Eq. 11 from 10 different parameter sets in Bojak and Liley (2005). These 10 sets are selected for the behavior of their α peak frequency under hyperpolarization, see text and Figures 3B,D.

Furthermore, if one makes the simplifying assumption that an EEG electrode aggregates the contributions of a disk-shaped part of the cortical sheet with radius *R*, then one can compute a prediction of the PSD as follows (Bojak and Liley, 2005)

(11) $$PSD\left(f\right)=2\pi R^{2}\overset{\infty }{\underset{0}{\int }}\frac{dk}{k}{\text{J}}_{1}^{2}\left(kR\right){\left|h_{e}\left(\omega \equiv 2\pi f,k\right)\right|}^{2},$$

where J_1_ is a Bessel function of the first kind. In practice we evaluate the integral Eq. 11 numerically using a 64 point 0 < *k_i_* < 14.14/cm Gauss–Legendre quadrature, and hence need to evaluate Eq. 10 for all these *k* = *k_i_*. PSDs calculated in this manner from 10 parameter sets selected out of the 73,454 in Bojak and Liley (2005) are shown in Figure 2. We call a solution *stable* if for all 64 *k* = *k_i_*, as well as for homogeneous cortex *k* = 0 cm, the eigenvalues are such that ∀*n*: Âλ*_n_*(*k*) < 0. Only for stable cases do all the approximations leading up to Eq. 11 make sense. The largest contributions to Eq. 10 arise when ω = ℑλ*_n_*(*k*); and if one disregards the *c_n_*(*k*), then the “least stable” eigenvalue with largest Âλ*_n_*(*k*) < 0 will contribute most.

Consider now only those λ*_m_* that have non-zero frequencies *f_m_* ≡ ℑλ*_m_*(*k* = 0/cm)/(2π) ≠ 0: due to the selection process (Bojak and Liley, 2005), the “least stable” λ_max_ of these λ*_m_* will have 8 Hz ≤ f_max_ ≤ 13 Hz, i.e., a frequency in the α rhythm range. If we change from parameter set {**P**_1_} to {**P**_2_}, we can compute the resulting frequency shift $\Delta f\equiv f_{\max}^{\left\{P_{2}\right\}}-f_{\max}^{\left\{P_{1}\right\}}$ of this eigenvalue α frequency. We find that for the parameter changes considered below, this “theoretical” α frequency shift Δ*f* estimated directly from the eigenvalues provides a reasonable approximation for a more “experiment-like” calculation of the α peak shift. In an experiment one would typically seek the maxima of the measured PSDs in the 8–13 Hz range, and then compute their difference in frequency in order to determine an α frequency shift, cf. Figure 1C (Hayashi et al., 2007; Tsuda et al., 2007). We can do something similar here by evaluating the full PSDs with Eq. 11 for {**P**_1_} and {**P**_2_}, respectively, and then compute the difference of the maxima of these theoretical predictions. However, we use the “theoretical” Δ*f* in the following. It is much easier to compute, since it involves only one eigendecomposition for *k* = 0/cm compared to 64 for *k* = *k_i_* needed in the numerical PSD integration Eq. 11. Furthermore, the “theoretical” Δ*f* separates the change of the α peak frequency from other changes to the spectrum. “Experiment-like” calculations of shifts directly from local maxima in the full spectrum can be confounded easily by other spectral changes, and a prior subtraction of the spectral “background” around these maxima would closely match our “theoretical” procedure. For the parameter sets shown in Figure 2, a comparison between “theoretical” and “experiment-like” frequency shifts is provided by Table 1. How these shifts are generated will be discussed in the following, but note for now that most results are quite similar. The big discrepancies for parameter set IX are caused precisely by a rise of the spectral “background,” as discussed.

**Table 1:** **α peak frequency shifts predicted from the leading eigenvalue (Δ*f*) and the full PSD, respectively, for the parameter sets of Figure 2**.

	Propofol only *P* = 1.2, *K* = 0		Ketamine only *P* = 0, *K* = 1.4		Both *P* = 1.2, *K* = 1.4	
	Δ*f* (Hz)	PSD (Hz)	Δ*f* (Hz)	PSD (Hz)	Δ*f* (Hz)	PSD (Hz)
I	0.25	0.07	−1.42	−1.70	1.84	1.85
II	0.40	0.29	−1.60	−1.69	2.70	2.61
III	0.24	0.13	−1.37	−1.69	1.91	2.27
IV	0.45	0.37	−1.10	−0.96	2.55	2.54
V	−0.20	−0.21	−1.39	−1.30	1.51	1.40
VI	0.12	−0.18	−1.00	−1.44	1.47	1.44
VII	0.10	0.16	−2.12	−2.11	2.72	2.81
VIII	0.02	−0.15	−1.33	−1.50	1.45	1.59
IX	0.20	−1.46	−1.89	−3.02	2.09	2.05
X	0.15	0.15	−1.38	−1.66	1.86	2.14

*P and K are normalized propofol and ketamine concentrations, respectively*.

### Drug effect parameterization and selection of sets

The effect of the action of both ketamine and propofol on HCN1 channels is to hyperpolarize the resting membrane potentials of pyramidal (excitatory) cells (Chen et al., 2009). Consider Eq. 6 in the absence of synaptic inputs *I_lk_*(**x**, *t*) ≡ 0, then $\lim_{t\to \infty }h_{k}(\text{x},t)=h_{k}^{r}$. Thus $h_{e}^{r}$ and $h_{i}^{r}$ parameterize the excitatory and inhibitory resting membrane potentials, respectively. In the spirit of Eq. 5 we hence use the following ansatz:

(12) $$\begin{array}{llll} \Delta h_{e}^{r}\equiv {\left.h_{e}^{r}\right|}_{P,K}-{\left.h_{e}^{r}\right|}_{P=K=0} & =-\left(a_{1}P+a_{2}K+a_{12}PK\right) & & \\ & =-\Delta h\cos\theta , \end{array}$$

(13) $$\begin{array}{llll} \Delta h_{i}^{r}\equiv {\left.h_{i}^{r}\right|}_{P,K}-{\left.h_{i}^{r}\right|}_{P=K=0} & =-\left(b_{1}P+b_{2}K+b_{12}PK\right) & & \\ & =-\Delta h\sin\theta , \end{array}$$

where *P*, *K* are normalized (dimensionless) concentrations of propofol and ketamine, respectively; and $\Delta h_{e}^{r},\Delta h_{i}^{r}$ are changes of the excitatory and inhibitory resting membrane potentials, respectively, due to these drugs. For convenience we have factored out the sign corresponding to hyperpolarization, and we have assumed that inhibitory neurons would react qualitatively like the pyramidal cells, i.e., $\Delta h_{e}^{r},\Delta h_{i}^{r}\leq 0\text{ mV}$ in the considered ranges 0 ≤ *P* ≤ *P*_max_ and 0 ≤ *K* ≤ *K*_max_, while quantitative differences are expressed by potentially different coefficients. Since the drugs applied individually lead to hyperpolarization, we must have coefficients *a*_1_, *a*_2_, *b*_1_, *b*_2_ > 0, whereas the sign of the interaction coefficients *a*_12_, *b*_12_ carries the same meaning as that of η in Eq. 4. In the following it often will be useful to express the “Cartesian” $\Delta h_{e}^{r},\Delta h_{i}^{r}\leq 0\text{ mV}$ in the corresponding “polar coordinate” form as $\Delta h\equiv \sqrt{{\left(\Delta h_{e}^{r}\right)}^{2}+{\left(\Delta h_{i}^{r}\right)}^{2}}\geq 0\text{ mV}$ and $\theta \equiv \arctan\frac{\Delta h_{i}^{r}}{\Delta h_{e}^{r}}\in \left[0^{\circ },90^{\circ }\right]$.

As a first step, we have investigated which of the 73,454 human α rhythm sets from Bojak and Liley (2005) can be extended viably via Eq. 12 and Eq. 13. Chen et al. (2009) found for rat pyramidal neurons that $\Delta h_{e}^{r}=-4.0\text{ mV}$ for ketamine at 20 μM concentration and $\Delta h_{e}^{r}=-3.7\text{ mV}$ for propofol at 5 μM concentration. Assuming that in humans (and in inhibitory neurons) hyperpolarizations of similar sizes occur, we varied both $h_{e}^{r}$ and $h_{i}^{r}$ away from their original values in steps of −0.05 mV up to a hyperpolarization of −6 mV, while the remaining parameters were left unchanged. This leads to a grid of 121 × 121 hyperpolarization combinations $\left(\Delta h_{e}^{r},\Delta h_{i}^{r}\right)$, for which we tested whether the changed parameter sets remain stable, i.e., we computed eigendecompositions for 64 + 1 values of *k* and made sure that all eigenvalues had negative real parts. We also calculated the resulting shift in the α peak frequency as compared to the original parameter set in the “theoretical” manner discussed above: $\Delta f\left(\Delta h_{e}^{r},\Delta h_{i}^{r}\right)\equiv f_{\max}^{\left\{P\left(\Delta h_{e}^{r},\Delta h_{i}^{r}\right)\right\}}-f_{\max}^{\left\{P\left(0,0\right)\right\}}$.

**Figure 3:**
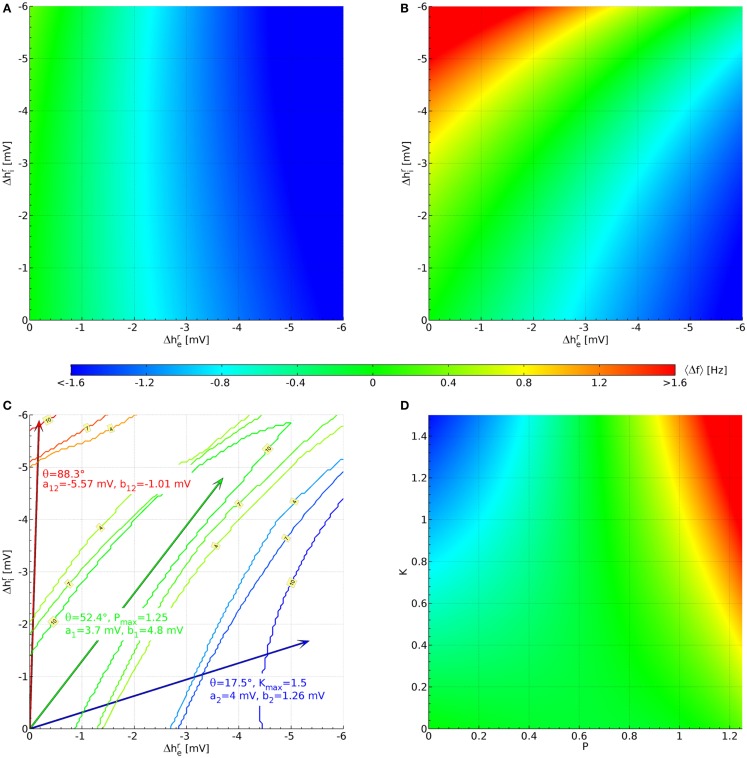
**Parameterization of the hyperpolarization effects of propofol and ketamine**. **(A)** Shift of the α peak frequency, average over all 1,627 sets estimated as described below Eq. 11. **(B)** Likewise, but averaged over the 10 sets shown in Figure 2, which were selected for having large up (Δ*f* > 1.6 Hz) and down (Δ*f* < −0.8 Hz) shifts of α peak frequency, as well as a lack of shift for some large hyperpolarizations (|Δ*f*(−3.7 mV, ≤4.3 mV)| < 0.4 Hz). **(C)** Blue contours indicate areas where 4, 7, or 10 sets have the required down-shift. A blue arrow points to the midpoint of this area, and drug effect parameters for Eq. 12 and Eq. 13 derived from this are listed in blue text. Likewise, parameters are derived for up-shift in red and a lack of shift in green. **(D)** These are the same results as in **(B)**, but now plotted against normalized propofol *P* and ketamine *K* concentrations using the drug effect parameters found in **(C)**.

We find that of the 73,454 parameter sets only 1,627 remain stable for all 121 × 121 combinations of hyperpolarizations up to −6 mV. This does not mean that the other parameter sets are thereby rejected on biological or physiological grounds; rather their PSDs cannot be calculated with the eigenspectrum approximation used here, but would have to be estimated from explicit simulations with the fully non-linear Eqs 6–8. This ordinary numerical procedure is several orders of magnitude slower and hence not employed here. Figure 3A displays the average $\left\langle \Delta f\left(\Delta h_{e}^{r},\Delta h_{i}^{r}\right)\right\rangle$ over the 1,627 stable sets. The color bar indicates the corresponding frequency values. We can see that in this average there is little effect of $\Delta h_{i}^{r}$, whereas decreasing $\Delta h_{e}^{r}$ (increasing the hyperpolarization of the pyramidal neurons) leads to an increasingly negative 〈Δ*f* 〉. The lowest average value for the 1,627 sets in Figure 3A is 〈Δ*f*(−6 mV, −6 mV)〉 = −2.03 Hz, whereas the highest is 〈Δ*f*(0 mV, −6 mV)〉 = 0.336 Hz.

Since more substantial increases in frequency are expected for the interaction of ketamine and propofol (Hayashi et al., 2007; Tsuda et al., 2007), we introduce the following cut: a set will be kept only if for at least one of the 121 × 121 hyperpolarization combinations $\left(\Delta h_{e}^{r},\Delta h_{i}^{r}\right)$ we find Δ*f* > 1.6 Hz. Similarly, since ketamine on its own should introduce a decrease in Δ*f*  (Schuttler et al., 1987; Kochs et al., 1996), we require that for at least one other hyperpolarization combination Δ*f* < −0.8 Hz. Finally, propofol on its own is assumed here to not change the α frequency significantly Δ*f* ≈ 0 Hz (Schwender et al., 1996; Kuizenga et al., 1998, 2001; Feshchenko et al., 2004; Breshears et al., 2010; Cimenser et al., 2011), at least not by a HCN1-mediated mechanism, as was discussed in the Introduction. It is more difficult to introduce a simple cut for this property, since for small hyperpolarizations by definition one finds Δ*f* ≈ 0 Hz. We orient ourselves here to $\Delta h_{e}^{r}=-3.7\text{ mV}$ for propofol from Chen et al. (2009), and require that at least for one combination with $\Delta h_{i}^{r}\leq -4.3\text{ mV}$ one has |Δ*f*(−3.7 mV, ≤4.3 mV)| < 0.4 Hz. Considered individually, the low frequency cut for ketamine eliminates only 80 parameter sets, whereas the high frequency cut for the interaction of ketamine and propofol leaves only 66 parameter sets. Combining these two cuts then leaves 64 parameter sets in total. Individually, the cut for propofol limiting the frequency shift leaves 149 parameter sets. Combined with the other two cuts, we arrive at 10 parameter sets. Their original PSDs are the ones that were displayed previously in Figure 2, and we display their parameter values in Table A1 in the Appendix. We show the resulting 〈Δ*f* 〉, now averaging over only the 10 selected sets, in Figure 3B. It is immediately apparent that there are now three zones: for small $\Delta h_{e}^{r}$ but large (negative) $\Delta h_{i}^{r}$ one sees large increases in frequency, for large (negative) $\Delta h_{e}^{r}$ but small $\Delta h_{i}^{r}$ large decreases in frequency, and in between there is a corridor with little change in frequency. This same basic structure is found in all 10 selected sets individually.

Now we can use this structure to determine the coefficients in Eq. 12 and Eq. 13. Starting with the case of giving ketamine only, we can write

(14) $$P=0:\tan\theta_{K}=\frac{b_{2}}{a_{2}}.$$

Thus the effect of increasing ketamine concentration in the plane of hyperpolarizations is to move out along a line through the origin with angle θ*_K_*. Ketamine on its own is supposed to deliver shifts to low frequencies, for which we have set a cut Δ*f* < −0.8 Hz above. We now determine for every hyperpolarization combination how many of the 10 selected parameter sets have $\Delta f\left(\Delta h_{e}^{r},\Delta h_{i}^{r}\right)<-0.8\text{ Hz}$. This leads to a 121 × 121 grid of values between 0 and 10. In Figure 3C this is shown by blue contour lines for 4, 7, and 10 sets fulfilling this cut. We choose the mean of all $\left(\Delta h_{e}^{r},\Delta h_{i}^{r}\right)$ in the “maximal fulfillment” (10 sets) region (tip of blue arrow) to determine tan θ*_K_* = 0.315. Given that we do not know the dependence of hyperpolarizations on ketamine concentrations in humans, we choose $\Delta h_{e}^{r}\equiv -4\text{ mV}$ at *K* = 1 and thus consequently *a*_2_ ≡ 4 mV. This implies an unknown normalization $K\equiv c_{K}∕c_{K}^{*}$, so that at a ketamine concentration $c_{K}^{*}$ one finds $\Delta h_{e}^{r}=-4\text{ mV}$ in humans. Given this choice, we have *b*_2_ ≡ 1.26 mV from the ketamine angle tan θ*_K_* = 0.315. Since *a*_2_ > *b*_2_, we can now also find *K*_max_ = (6 mV)/*a*_2_ = 1.5 as the largest value for the normalized ketamine concentration for which both hyperpolarizations remain below −6 mV.

In a similar manner we can deal with the case of propofol as the sole drug. Then we find the angular dependence:

(15) $$K=0:\tan\theta_{P}=\frac{b_{1}}{a_{1}}.$$

Figure 3C shows contour lines for 4, 7, and 10 parameter sets fulfilling the cut for an α frequency shift $\left|\Delta f\left(\Delta h_{e}^{r},\Delta h_{i}^{r}\right)\right|<0.4\text{ Hz}$, this time in green color. Since the cut was evaluated for $\Delta h_{e}^{r}=-3.7\text{ mV}$ only to find these sets, we determine the mean of combinations $\left(\Delta h_{e}^{r}\equiv -3.7\text{ mV},\Delta h_{i}^{r}\right)$ that have “maximal fulfillment” (10 sets) in order to obtain tan θ*_P_* = 1.297, indicated by the tip of the green arrow. We choose $\Delta h_{e}^{r}\equiv -3.7\text{mV}$ at $P\equiv c_{P}∕c_{P}^{*}=1$, so that *a*_1_ ≡ 3.7 mV and at an unknown propofol concentration $c_{P}^{*}$ one finds $\Delta h_{e}^{r}=-3.7\text{ mV}$ in humans. Then *b*_1_ ≡ 4.8 mV, and since *b*_1_ > *a*_1_ it follows that *P*_max_ = (6 mV)/*b*_1_ = 1.25.

Finally, Figure 3C shows red contour lines for 4, 7, and 10 parameter sets fulfilling $\Delta f\left(\Delta h_{e}^{r},\Delta h_{i}^{r}\right)>$1.6 Hz. Again we find the mean of “maximal fulfillment” (10 parameter sets), as indicated by the tip of the red arrow. These mean values are $\Delta {\bar{h}}_{e}^{r}\equiv -0.177$ {mV} and $\Delta {\bar{h}}_{i}^{r}\equiv -5.903$ mV in this case. We now extend to the −6 mV hyperpolarization limit by setting $\Delta {\bar{h}}_{i}^{\max}\equiv -6$ mV and $\Delta {\bar{h}}_{e}^{\max}\;\equiv$ (−6 mV)(−0.177 mV)/(−5.903 mV) = −0.180 mV. We can now solve the following two equations

(16) $$\begin{array}{l} \Delta {\bar{h}}_{e}^{\max}=-\left(a_{1}P_{\max}+a_{2}K_{\max}+a_{12}P_{\max}K_{\max}\right), \end{array}$$

(17) $$\begin{array}{ll} \Delta {\bar{h}}_{i}^{\max}=-\left(b_{1}P_{\max}+b_{2}K_{\max}+b_{12}P_{\max}K_{\max}\right). & \end{array}$$

This will then mean that our entire hyperpolarization grid $-6\text{ mV}\leq \Delta h_{e}^{r},\Delta h_{i}^{r}\leq 0\text{ mV}$ will be projected onto a rectangular area bounded by 0 ≤ *P* ≤ *P*_max_ and 0 ≤ *K* ≤ *K*_max_, respectively. Solving Eq. 16 and Eq. 17 with our previous results yields *a*_12_ = −5.57 mV and *b*_12_ = −1.01 mV. Figure 3D shows the projected 〈Δ*f*(*P*, *K*)〉. Clearly the intended α frequency shifts are now achieved: negative ones for only ketamine, none for only propofol, and positives ones for propofol and ketamine together.

## Results

We have parameterized the HCN1-mediated hyperpolarizations of neuron membrane potentials in order to reproduce the observed EEG effects of ketamine and propofol, and in particular of their interaction when concurrent. The coefficients that we have obtained for Eqs 12–13 afford the following interpretation: pyramidal neurons react similarly to ketamine and propofol (*a*_1_ = 0.925 × *a*_2_), whereas inhibitory neurons react much more strongly to propofol than to ketamine (*b*_1_ = 3.81 × *b*_2_). Furthermore, and perhaps most interestingly, there is an antagonism of ketamine and propofol (*a*_12_, *b*_12_ < 0), which leads to infra-additivity in the investigated effect of HCN1-mediated hyperpolarization, cf. Eq. 4. This antagonism is stronger in pyramidal neurons *a*_12_/(*a*_1_*P*_max_ + *a*_2_*K*_max_) = 4.10 *b*_12_/(*b*_1_*P*_max_ + *b*_2_*K*_max_), though the precise proportion depends on the given concentrations of the drugs. Intuitively it makes sense however that in inhibitory neurons, where one drug is much more effective than the other, the antagonism between the drugs is less pronounced.

**Figure 4:**
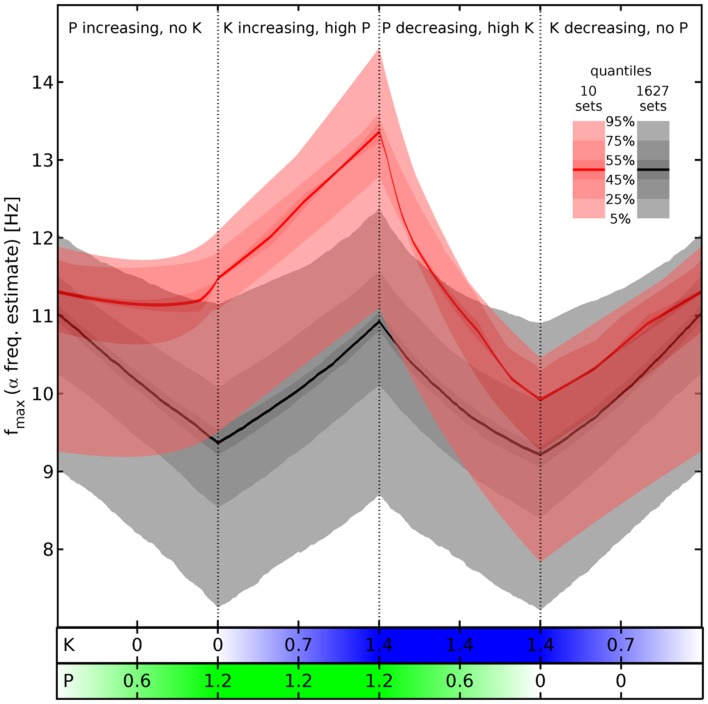
**Estimated α peak frequency shifts**. Shifts of the α peak frequency for normalized propofol *P* and ketamine *K* concentrations estimated as described below Eq. 11, using the hyperpolarizations in Eq. 12 and Eq. 13. Either all 1,627 (gray) or the 10 selected sets (red) are used to compute quantile bands, as indicated by the legend. The median value is shown by a thick black or red line, respectively. There are four phases of drug variation, as indicated by the titles and dotted lines, quantified by bars below the main panel: first, *P* = 0 → 1.2 linearly, while *K* = 0. Then *K* = 0 → 1.4 linearly, while *P* = 1.2. Next *P* = 1.2 → 0, while *K* = 1.4. Finally, *K* = 1.4 → 0, while *P* = 0. No pharmacodynamics has been modeled here, so every (*P*, *K*) combination yields an independent “steady state” result. Hence for example an increase of *P* at high *K* is shown by the third phase viewed from right to left.

To illustrate these results we look again at the “theoretical” estimates of the α peak frequency in Figure 4, where we compare now the effects of changing propofol and ketamine concentration on the 10 selected sets (red) with those computed for all the valid 1,627 sets (gray). Note that the 1,627 sets include the 10 selected ones. Quantile bands are computed to summarize the results for the individual parameter sets, as indicated by the legend. Starting from a baseline without drugs, four phases are being considered: first, propofol concentration is increased linearly; then propofol is maintained at maximum concentration and ketamine concentration is increased linearly; next propofol concentration is decreased linearly while ketamine is maintained at maximum concentration, and finally ketamine concentration is decreased linearly for a return to the baseline. It should be noted that no attempt at modeling the pharmacodynamics/pharmacokinetics of ketamine and propofol drug action beyond drug interaction has been made here. Furthermore, the eigenspectrum approach assumes that the system has reached equilibrium for the given parameters. Thus every single *f*_max_(*P*, *K*) predicted here, and consequently every single quantile band value, represents a “steady state” result for that particular drug concentration combination. Hence one can for example view Figure 4 from right to left, beginning with an increase in ketamine concentration, followed by an increase in ketamine at maximum propofol concentration, and so forth.

Comparing now the red with the gray quantile bands, we see that our cuts selected sets that react particularly dramatically to the concurrence of propofol and ketamine (phases 2 and 3), while being unresponsive to propofol alone (phase 1). Nevertheless, it is not the case that the results for the 1,627 sets show a totally divergent response pattern. In fact, the median rise of estimated α peak frequency in phase 2 (upon introducing ketamine at maximum propofol concentration) is comparable: 1.88 Hz for the selected sets (from 11.48 to 13.36 Hz) vs. 1.56 Hz for all sets (from 9.37 to 10.93 Hz). Thus the predicted boost of α peak frequencies due to the interaction between ketamine and propofol is a robust result for all sets given our drug effect parameterization, which is infra-additive concerning HCN1-mediated hyperpolarization. The main difference appears to be rather that the α peak frequencies of the selected sets do not react significantly to propofol, whereas they are similar to all other sets in the reaction to ketamine and the interaction between these drugs.

**Figure 5:**
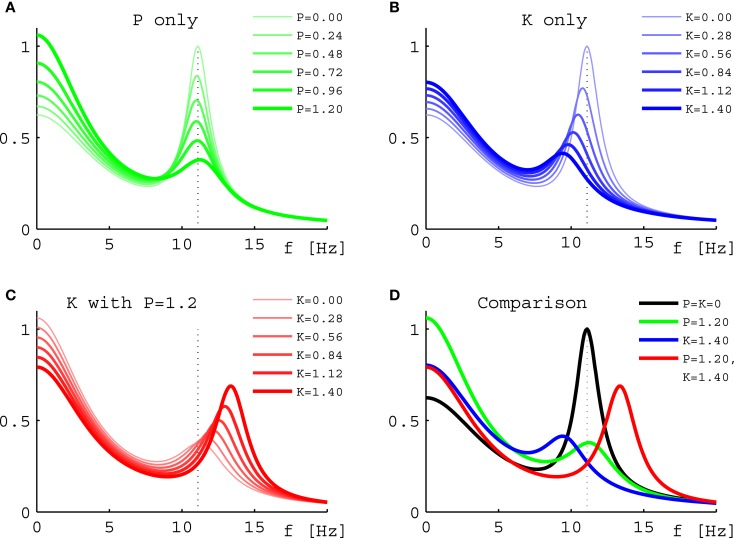
**Power spectral densities for Set III under drug variations**. **(A)** PSDs for increasing normalized propofol concentration from none (thinnest green line) to 1.2 (thickest green line). **(B)** PSDs for increasing normalized ketamine concentration from none (thinnest blue line) to 1.4 (thickest blue line). **(C)** PSDs for increasing normalized ketamine concentration from none (thinnest red line) to 1.4 (thickest red line), while normalized propofol concentration is held constant at 1.2. **(D)** Comparison of the PSDs representing the highest normalized concentrations from **(A)** in green, **(B)** in blue, and **(C)** in red. The black curve is the PSD without drugs. In all four panels the dotted line represents the position of the α peak of this curve.

Turning to results for full PSDs from Eq. 11, we will consider the 10 selected sets only due to the higher computational demands. Figure 5 shows results for one individual set (Set III of Figure 2) under three variations of drug concentration. In Figure 5A we see that as desired and estimated, the α peak frequency stays roughly the same during propofol anesthesia (Schwender et al., 1996; Kuizenga et al., 1998, 2001; Feshchenko et al., 2004; Breshears et al., 2010; Cimenser et al., 2011). The damping seen here would be more characteristic of occipital than frontal regions, though other processes in particular related to the GABA_A_ agonism could modify these results. The “theoretical” Δ*f* = 0.24 Hz at *P* = 1.2 is larger than the “experiment-like” shift of 0.13 Hz. In Figure 5B we can see the reaction to increasing ketamine concentration. As expected, the α peak gets shifted to lower frequencies. The “theoretical” Δ*f* = −1.37 Hz estimate at *K* = 1.4 is somewhat lower than the “experiment-like” shift of the local maxima of the PSDs of −1.69 Hz. While the α oscillations get dampened, they contribute to a net increase in the θ frequency range (Schuttler et al., 1987; Kochs et al., 1996) thanks to their downward frequency shift. But one sees also a general rise in power at lower frequencies. Figure 5C shows that adding ketamine in the presence of high doses of propofol leads to a shift of the α peak into the beta band, as observed by Hayashi et al. (2007) and Tsuda et al. (2007). The “theoretical” Δ*f* = 1.91 Hz at *P* = 1.2 and *K* = 1.4 is smaller than the “experiment-like” shift of 2.27 Hz. We predict here an increase in power, unlike the experiment, which observed a small but significant reduction, and our frequency shift of 2.27 Hz is less than half the observed 4.7 Hz. But this could be explained easily by the missing GABA_A_ and NMDA mechanisms, or the precise circumstances of the experiment. A bolus of ketamine was given by intravenous injection in the experiment, whereas here we calculate “steady state” results. Furthermore, while the frequency shifts from our “theoretical” Δ*f* method are largely in agreement with those obtained from the local α maxima of the PSDs, they do not agree perfectly – in spite of both being derived using the same eigenspectrum technique. The difference is that by looking at the maxima the results are influenced by changes to the overall spectrum, which provide the “background” on which the α resonance sits. Table 1 gives Δ*f* and “experiment-like” α peak frequency shifts for the 10 sets at the three highlighted drug concentrations.

**Figure 6:**
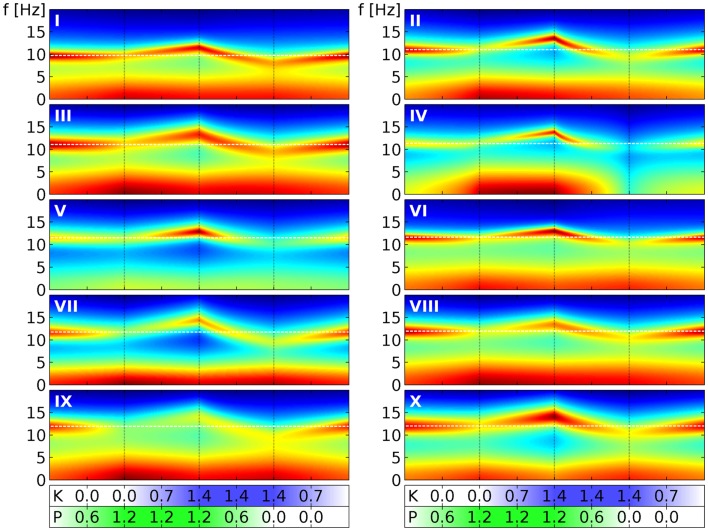
**Power spectral densities for all 10 selected parameter sets under drug variation**. We use here the same four phases of drug variation as in Figure 4, as indicated by the dotted lines and quantified by bars below the main panels: first, *P* = 0 → 1.2 linearly, while *K* = 0. Then *K* = 0 → 1.4 linearly, while *P* = 1.2. Next *P* = 1.2 → 0, while *K* = 1.4. Finally, *K* = 1.4 → 0, while *P* = 0. Every panel corresponds to 1 of the 10 selected parameter sets, as indicated by a white roman numeral. The PSD for one specific (*P*, *K*) combination is indicated in the panel by a colored vertical line corresponding to frequencies from 0 to 20 Hz. Colors here indicate decibels of the PSD, with dark red corresponding to large, green to medium and dark blue to small values. (The “jet” colormap of Matlab has been mapped for each panel individually, to the full range of PSD decibel values shown in the panel.) A white dashed line indicates the α peak frequency in the absence of drugs.

Finally, in Figure 6 we show similar results for all the 10 selected sets. We follow here the same scheme of changing drug concentrations as in Figure 4. We see that the α peaks of the full PSDs (here shown in decibels by color) of the individual sets indeed follow the “zigzag” shape we saw in the quantile bands of Figure 4. Panel III in Figure 6 can be directly compared to Figure 5, which we have just discussed. For example, Figure 5A corresponds to the first phase in panel III here. Overall we see that while the sets clearly change in a similar way, they all have individual features that set them apart from the others. For example, parameter set IV shows particularly strong changes in the low frequency range, whereas parameter set VII reacts with a particularly strong lowering of the α peak frequency in the presence of ketamine. These variations can be considered as representing the variations that one can also observe in humans.

## Discussion

We have shown that observed changes of the EEG α peak frequency induced by the presence of the anesthetic agents propofol and ketamine, but in particular also by their interaction when given concurrently, can be explained based on the modeling of HCN1-mediated hyperpolarizations alone, at least qualitatively. This is surprising, since the main mechanism of action of these drugs is supposed to be through NMDA antagonism (ketamine) and GABA_A_ agonism (propofol), respectively. However, since HCN1-knockout mice are indeed less sensitive to the hypnotic effects of both drugs (Chen et al., 2009), this would indicate that the EEG remains useful as an indicator of anesthetic action. It is perhaps interesting to note that while ketamine is famous for its hallucinatory action (Wolff and Winstock, 2006), and hence is considered a dissociative anesthetic agent, propofol as a classic general anesthetic agent is also capable of inducing a range of hallucinatory phenomena (Balasubramaniam and Park, 2003). Hence it is possible that psychotropic HCN1-mediated effects are simply masked behaviorally more by propofol’s GABA_A_ agonism than by ketamine’s NMDA antagonism, but that the EEG is particularly sensitive to these underlying changes.

Only a fraction of all considered parameter sets (1,627 of the 73,454 parameter sets from Bojak and Liley (2005) proved “stable” under the HCN1-mediated hyperpolarization changes up to −6 mV on both excitatory and inhibitory neurons. However, this is at least partly due to the computational methods used here: the eigenspectrum method (Bojak and Liley, 2005) can only be used for “fixed point” dynamics. Rejected sets could follow the same kind of drug-induced changes, but be inaccessible with our method chosen for its computational speed. In principle it would be desirable to carry out fully non-linear calculations instead, but under extra-cortical noise input it takes about a minute to estimate a sufficiently detailed and accurate power spectrum on a regular PC. For the 121 × 121 hyperpolarization changes of Figure 3 that would lead to about 10 days of calculations even for a single parameter set – but we investigate here 73,454 different ones. The eigenspectrum method is several orders of magnitude faster even for a single neural mass. Yet we include here also effects due to the spatial distribution of neural masses, see Eq. 11. The computational load scales roughly linearly with the number of integration points for the eigenspectrum method, but roughly as a square for an equivalent fully non-linear simulation based on spatial grids. This increases the difference in computation speed even further. Thus for the investigations carried out here only the eigenspectrum method proves practicable. In addition, limit cycle or chaotic dynamics are often more representative of seizures or other pathological brain states, which in clinical practice would lead to the termination of the pharmacological intervention that we intend to describe here. Other than by numerical simulation in every single case, we do not know how to determine the characteristics of the dynamics past the point of instability.

Furthermore, our current investigation does not include the NMDA and GABA_A_ actions commonly assumed to be dominant in these drugs. We speculate that a more complete simulation could allow the use of a larger fraction of the 73,454 parameter sets, since these omitted actions can affect the required stability. A prolongation of the inhibitory postsynaptic potentials due to GABA_A_, for example, could suppress excessive excitation and thus stabilize a parameter set. These neglected stabilizing effects would increase also in due proportion to the agent concentration, just as the potentially destabilizing hyperpolarizations we have modeled here do. In order to obtain spectral changes that demonstrate clearly the expected frequency shifts, we introduced three further selection cuts, leaving us with only 10 parameter sets out of the 1,627. Again we speculate that NMDA and GABA_A_ actions may ameliorate this reduction. If this is not the case, then this may point to underlying correlations between neural parameters or functional properties that were not considered in Bojak and Liley (2005), but which now prove crucial for a realistic description. Note that in terms of the hyperpolarizations, cf. Figures 3B,C, the non-reactivity to propofol means that for the 10 selected parameter sets an increase in the hyperpolarization of excitatory neurons can be compensated by an increase in the hyperpolarization of inhibitory neurons. This could suggest a particular intrinsic balance of excitation and inhibition maintaining functional stability against extrinsic disturbances.

As is apparent from Figure 2, most of our 10 selected sets have relatively high α peak frequencies. However, this simply reflects an underlying bias in the original 73,454 parameter sets, cf. Figure 8 in Bojak and Liley (2005); and we were able to lower base α frequencies through the adjustment of a few parameters (e.g., cortico-cortical connectivity $N_{lk}^{\beta }$) within physiological limits, without thereby qualitatively changing the relative frequency shifts due to propofol and ketamine. Other spectral features that could be selected for are also unlikely to affect the α frequency shifts here qualitatively. The constraints used by Bojak and Liley (2005) leave plenty of room for such adjustments: effectively only four out of 14 system eigenvalues were used to establish the “1/*f*” background and the α resonance. Adding a weak beta frequency resonance to enhance realism, for example, would only require the adjustment of two further system eigenvalues. Thus we expect that our results here would hold true if one were to redo the parameter space search of Bojak and Liley (2005) first with additional constraints on the base power spectra. Finally, for concurrent application of propofol and ketamine we predict increases of the α peak frequency of around 2 Hz, falling short of the 4.7 Hz seen experimentally (Hayashi et al., 2007; Tsuda et al., 2007). Yet our calculations were for “steady state” concentrations, and for rapid increases in dosage, as in the experimental injection of a single ketamine bolus here, one often finds a more complex response due the pharmacodynamics and transient neural responses. The so-called biphasic responses to anesthetic agents (Kuizenga et al., 1998, 2001) has received theoretical attention from several groups (Bojak and Liley, 2005; Wilson et al., 2006; Molaee-Ardekani et al., 2007; Hutt and Longtin, 2010; Steyn-Ross and Steyn-Ross, 2010), see also the review in Foster et al. (2008). Hints of such a biphasic response could be visible in Figure 5 of Tsuda et al. (2007), which shows a significant drop of the α peak shift with time from the initial 4.7 Hz to values around 2 Hz. However, this would have to be disentangled from the decrease in ketamine concentration due to natural clearance after the bolus. We intend to investigate all the mentioned issues in future work.

We found that we could account for the heterogeneous effects of ketamine on the EEG if we assumed that propofol and ketamine interacted in an *infra-additive* *or antagonistic* manner in their inhibition of HCN1-mediated neuronal membrane hyperpolarization. While most anesthetic and sedative agents are reported to interact synergistically ketamine is well-known to be a major exception (Hendrickx et al., 2008). The interaction between ketamine and GABA_A_ agonists (most sedative/anesthetic agents) to produce hypnosis is reported to range from additivity to infra-additivity/antagonism (Hendrickx et al., 2008). On the basis of the limited clinical data available it appears that the GABA_A_ agonist propofol can interact infra-additively to produce a given hypnotic endpoint. For instance Hui et al. (1995), in a study involving 180 female patients presenting for minor gynecological surgery, calculated quantal dose-response curves for propofol and ketamine administered alone and in combination. On the basis of logarithmic regression of a response surface model, it was first suggested that the dose-response for the combination was best explained by additivity, but on reanalysis (Hendrickx et al., 2008), by infra-additivity (significantly so for immobility, as trend for hypnosis). In apparent contradiction, Sakai et al. (1999) claim a significantly additive interaction between propofol and ketamine for the endpoints of unresponsiveness to vocal command and the loss of eyelash reflex. However, the former study determined the probability of a given hypnotic endpoint in response to *single doses* of fixed proportions of ketamine and propofol, whereas the latter gave *continuous infusions* of ketamine and propofol until a given hypnotic endpoint was reached. Another study by Frizelle et al. (1997) used smaller doses of ketamine and targeted a lower level of sedation (rousable to verbal stimuli) with a bolus of propofol and ketamine followed by concomitant infusion. They found no statistical evidence that the addition of ketamine reduced the required amount of propofol to reach their intended sedation level, suggesting once more infra-additivity. Clearly, additional work regarding the pharmacodynamic interactions of ketamine and propofol are required, as it is difficult to reconcile the results of these studies.

The use of neural field/mass approaches to modeling drug action on the EEG is emerging as a powerful explanatory framework (Liley and Bojak, 2005; Foster et al., 2008; Hutt, 2011), which is able to retain meaningful connections to the brain’s physiology despite its mesoscopic scale of description. Because neural field/mass models generally have much smaller parameter and state spaces than biophysically plausible neural network models, they are not only much easier to parameterize and simulate, but offer a simpler framework from which to make predictions and derive hypotheses that can be empirically tested. Our ability to account qualitatively for the effects that propofol and ketamine have on the EEG adds to a growing list of phenomena that are amenable to neural field/mass description (Deco et al., 2008; Coombes, 2010; Bressloff, 2012; Liley et al., 2012). Ultimately it is hoped that by accounting for large scale phenomena using neural field/mass models, genuine and enduring insights into brain function and physiology will emerge.

## Conflict of Interest Statement

David T. J. Liley is Chief Scientific Officer of Cortical Dynamics Ltd., an unlisted subsidiary of Biopharmica Ltd. (Perth, Australia), which is a medical device company focused on developing an EEG based depth of anesthesia monitor. David T. J. Liley is an inventor on several patent applications filed by Cortical Dynamics Ltd. since 2004 that describe new approaches to monitoring depth of anesthesia using the EEG. None of the IP declared in any published, pending, or granted patent has been licensed.

## Appendix

**Table A1:** **The 10 selected parameter sets, whose PSDs are shown in Figure 2**.

	I	II	III	IV	V	VI	VII	VIII	IX	X
$h_{e}^{r}$ (mV)	−68.718	−70.286	−69.774	−78.169	−63.407	−60.745	−67.15	−64.128	−64.061	−60.588
$h_{i}^{r}$ (mV)	−71.115	−78.148	−69.325	−79.978	−72.375	−70.488	−79.864	−79.399	−70.777	−69.941
τ*_e_* (ms)	149.16	83.072	125.02	104.53	69.026	86.932	134.75	77.855	96.538	109.63
τ*_i_* (ms)	125.78	122.72	116.58	112.75	118.65	50.200	66.766	137.77	43.662	76.350
$h_{ee}^{\text{eq}}$ (mV)	1.8642	−16.433	3.2177	8.7034	−2.5551	1.9520	−18.571	−9.8354	8.4179	0.83573
$h_{ei}^{\text{eq}}\left(mV\right)$	−13.716	5.2227	8.2845	−14.231	−17.725	−15.828	−19.572	−4.4417	4.0220	2.4429
$h_{ie}^{\text{eq}}\left(mV\right)$	−86.369	−85.969	−86.775	−86.941	−85.466	−83.939	−86.449	−87.012	−82.833	−87.292
$h_{ii}^{\text{eq}}$ (mV)	−80.439	−85.348	−77.200	−86.445	−83.047	−79.708	−87.791	−87.906	−78.523	−78.755
Γ*_ee_* (mV)	0.22666	0.15856	0.17189	0.11073	0.25964	0.18606	0.31401	0.11187	0.10671	0.15192
Γ*_ei_* (mV)	0.72933	1.8661	1.7385	1.8429	1.7030	0.91706	1.7073	1.2797	0.60619	1.7838
Γ*_ie_* (mV)	1.9579	1.6800	1.5436	1.6612	1.8285	1.1699	0.53775	1.2751	0.31684	1.2942
Γ*_ii_* (mV)	1.0898	0.68575	0.61488	0.53105	0.87001	0.38026	0.10299	0.59535	0.38033	1.2539
γ*_ee_* (s^−1^)	768.09	979.20	626.91	494.19	399.13	848.11	964.40	795.80	689.20	355.38
γ*_ei_* (s^−1^)	128.26	399.71	357.24	170.95	246.39	219.24	238.50	191.63	224.39	258.87
γ*_ie_* (s^−1^)	192.29	178.41	135.36	411.10	251.74	82.043	468.53	221.95	133.21	203.10
γ*_ii_* (s^−1^)	57.060	58.091	52.773	53.437	49.217	50.113	43.132	57.302	59.581	49.348
$N_{ee}^{\beta }$	3393.0	2552.5	2337.7	4557.9	3571.9	2945.1	2002.9	3941.9	2061.2	2718.0
$N_{ei}^{\beta }$	4520.7	4183.2	4168.8	4922.0	4290.5	2771.2	3297.8	4833.0	4357.1	4964.4
$N_{ie}^{\beta }$	270.31	674.75	566.64	934.52	927.91	520.26	703.66	838.55	835.88	607.11
$N_{ii}^{\beta }$	125.69	453.59	594.80	141.53	472.96	658.36	294.98	890.80	314.72	147.19
$N_{ee}^{\alpha }$	4223.4	2234.3	4974.3	2517.6	2871.9	2230.2	2678.3	2874.7	4781.7	4128.1
$N_{ei}^{\alpha }$	2892.3	1559.1	2837.9	2412.1	2952.9	1441.3	1693.6	2896.0	2095.3	2078.7
Λ (cm^−1^)	0.69280	0.27742	0.2529	0.76041	0.16148	0.22388	0.84812	0.56276	0.83448	0.51625
ν (cm^−1^)	116.05	137.60	483.12	158.20	156.22	283.76	483.97	504.36	790.03	325.52
$S_{\text{e}}^{\max}$ (s^−1^)	311.08	201.57	474.21	126.59	88.686	103.54	280.25	422.80	190.77	246.41
$S_{\text{i}}^{\max}$ (s^−1^)	249.73	280.48	287.93	171.23	227.44	238.74	473.82	294.99	485.67	411.64
${\bar{\mu }}_{e}$(mV)	−45.365	−49.195	−53.432	−54.004	−44.616	−46.851	−46.811	−43.850	−47.622	−51.391
${\bar{\mu }}_{i}$ (mV)	−49.046	−45.093	−51.480	−44.165	−48.954	−47.996	−51.395	−50.619	−42.285	−44.831
${\hat{\sigma }}_{e}$(mV)	6.5908	6.7284	5.2051	6.8209	6.9030	5.9824	5.9045	6.6268	5.6959	5.0919
${\hat{\sigma }}_{i}$ (mV)	4.3224	4.7270	4.4501	4.1542	5.4314	3.0605	5.4229	5.8536	5.6195	6.6326
${\bar{p}}_{ee}$ (s^−1^)	7795.9	7344.6	7966.7	5876.2	4496.4	3882.5	6781.8	2649.9	9342.5	2833.6
$p_{ei}$ (s^−1^)	329.39	2554.0	999.87	2120.3	2188.8	2337.9	1196.8	2063.5	914.37	1339.4
